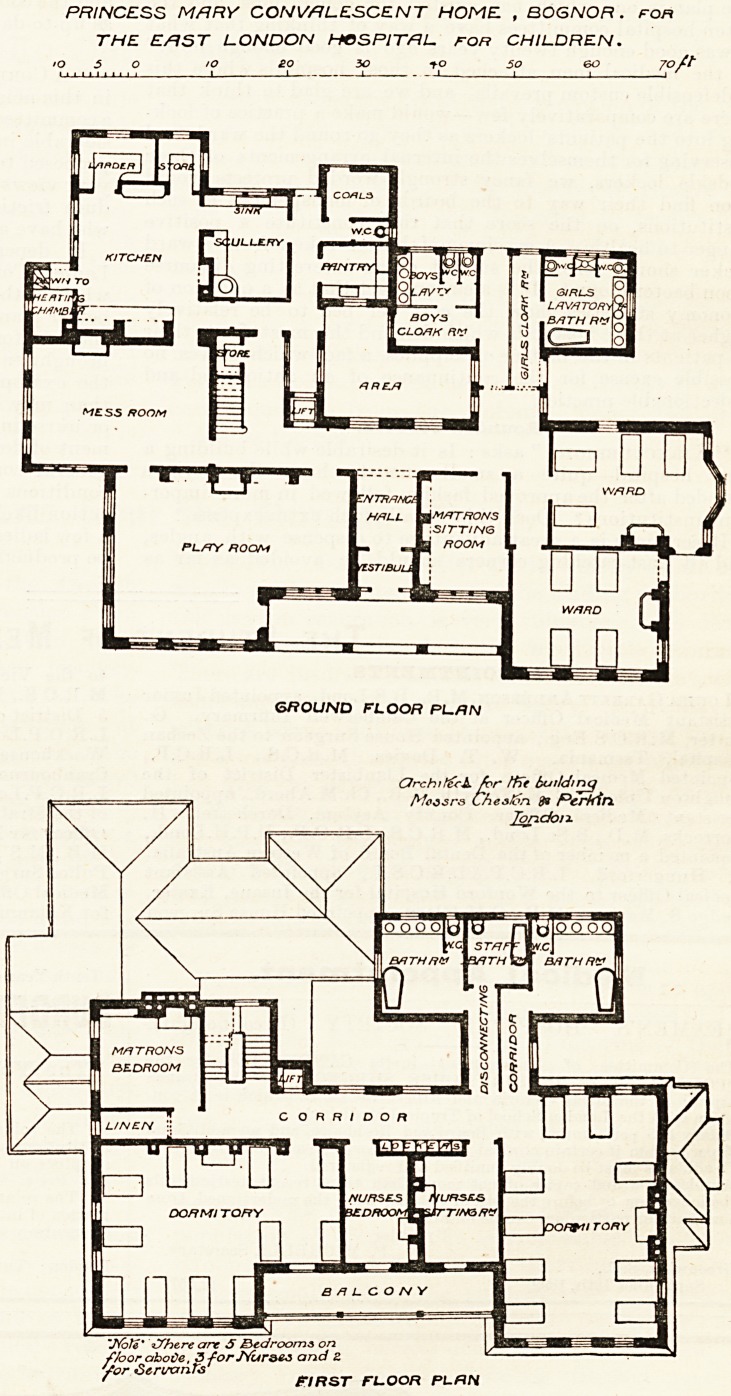# Hospital Construction

**Published:** 1899-09-30

**Authors:** 


### HOSPITAL CONSTRUCTION.

### THE PRINCESS MARY CONVALESCENT HOME, BOGNOR.

THIS convalescent home, as will be seen from the plans we publish herewith, is designed on the villa idea. We should have preferred a nearer approach to the hospital form of construction; but apart from this objection we notice much to admire. Presumably this building has a southern exposure, but we cannot positively say so as the points of the compass are not marked on the plan supplied to us. The main entrance is prettily arranged under a verandah, and a vestibule divides this from the hall. On the right of the hall on entering is the matron's sitting-room, and further to the right are two small dormitories of four beds each. These are intended for surgical cases, and it is here that we think the pavilion form of ward would have been so much better for the purpose. Behind these are the boys' lavatories and closets and the girls' lavatories and closets. This small block is carefully cut off from the main building by a ventilating passage, and the boys and girls reach their lavatories by separate corridors. On the left of the hall is the play-room. It has a door leading to the verandah and another leading to the mess-room, which room is placed immediately behind the play-room. Here, again, although the objection may not be so great as with the dormitories, we should like to have seen a room projecting from the main block, and having windows on both sides and on its free end. Our experience has been that such rooms as are shown on the plan are very apt to become "stuffy." Beyond the mess-room are the kitchen and various offices, and the former has a swing door leading to the mess-room. On the first floor and to the right are the nurses' sitting-room, and a dormitory for nine beds. The latter is the best planned room in the house. To the rear of these are the lavatories and bath-rooms, which are, of course, placed over those on the ground floor, and, like the others, have ventilating passages. To the left is a dormitory for eleven beds. The matron's bed-room is behind the dormitory. The second floor gives accommodation for three nurses and two servants. The kitchen and kitchen offices are not carried up beyond the first storey. The home is built of Tortington bricks, and the outer walls are hollow, except where the outer aspect is protected by tiles. The floors of the lavatories and bath-rooms are of concrete covered with mosaic. The drainage was superintended by Mr. Tyndale, sanitary engineer to the War Office. The drainage pipes are jointed with cement, and laid on concrete.

PRINCESS MARY CONVALESCENT HOME, BOGNOR. FOR THE EAST LONDON HOSPITAL FOR CHILDREN.

GROUND FLOOR PLAN

FIRST FLOOR PLAN

## Figures and Tables

**Figure f1:**